# A Questionnaire of Physiotherapeutic Specific Exercises of Scoliosis—QPSSE

**DOI:** 10.3390/jcm13020318

**Published:** 2024-01-05

**Authors:** Theodoros B. Grivas, Dimitra Dadakaridou, Stavroula A. Fokidi, Alexandros Kastrinis, Melpomeni Kosti, Constantinos Mihas, Marianna Oikonomaki, Evangelos Theodosopoulos, Artemis Margarita Griva

**Affiliations:** 1 Department of Orthopaedics & Traumatology, “Tzaneio” General Hospital of Piraeus, 18536 Piraeus, Greece; 2 Human Performance Lab, Department of Physical Education & Sports Science, Aristotle University of Thessaloniki, 54124 Thessaloniki, Greece; dadadimi@phed.auth.gr; 3 Department of ICU, “Agios Panteleimon” General Hospital of Nikea, 18454 Piraeus, Greece; stav_fokidi@yahoo.gr; 4 Health Assessment and Quality of Life Research Laboratory, School of Health Sciences, Physiotherapy Department, University of Thessaly, 3rd km Old National Road Lamia-Athens, 35132 Lamia, Greece; alexkastrinis@uth.gr; 5 Physiotherapist & Complementary Therapist (Acupuncture), 15123 Athens, Greece; melpo-kosti@hotmail.com; 6 Department of Internal Medicine, Kymi General Hospital—Health Centre, 34003 Evia, Greece; gas521@yahoo.co.uk; 7 Aenaon-Physio, 17342 Athens, Greece; mariannaoikonomaki@gmail.com; 8 Athens Scoliosis-Spine Rehabilitation Clinic, Moschato, 18345 Athens, Greece; athensscoliosis@gmail.com; 9 Department of Social Psychology, Neapolis University Pafos, 2 Danais Avenue, 8042 Pafos, Cyprus; a.griva@nup.ac.cy

**Keywords:** adolescent idiopathic scoliosis, Physiotherapeutic Specific Exercises, questionnaire, quality of life

## Abstract

Study design: Creating and psychometric testing of a new QoL Questionnaire about Physiotherapeutic Specific Exercises of Scoliosis (Questionnaire of Physiotherapeutic Specific Exercises of Scoliosis—QPSSE). Purpose: The purpose of this study is to create a reliable and valid questionnaire for patients suffering from mild and moderate adolescent idiopathic scoliosis (AIS) who have been treated with Physiotherapeutic Specific Exercises of Scoliosis (PSSE) in order to evaluate their quality of life. Materials and Methods: The developed questionnaire was based on a thorough literature review as well as on authors’ experience. It consists of 53 questions, of which 37 have a positive meaning, 15 have a negative meaning, and 1 is a multiple choice question; additionally, there are 6 “open” questions. Except for the multiple choice question, all other questions are answered on a Likert scale ranging from 1 to 5 points. Five represents a positive meaning or very positive one, whereas one stands for a negative meaning or none at all. Questions were developed by the authors who subsequently categorized the 53 questions into the following eight domains: physical functioning, self-image, Physiotherapeutic Scoliosis-Specific Exercises (PSSEs), psychosocial functioning, cognitive functioning, compliance, motivation, and pain. A pilot study was conducted so that we could calculate Cronbach’s Alpha based on the outcome. Due to the COVID-19 pandemic, the authors worked through the Zoom online platform to structure the questionnaire. Results: Pearson’s correlation coefficient was used for all correlations evaluated. P values of less than 0.05 were considered to be significant. Internal consistency was evaluated with Cronbach’s Alpha. Although there were very few missing values, accounting for 0.78% of the total values of the questionnaire, the expectation maximization likelihood algorithm was used to impute data. IBM^®^ SPSS^®^ Statistics Software v.25 was used for the analysis. Cronbach’s Alpha coefficients for the overall score were 0.84. Conclusions: This original QPSSE was found to be a reliable and valid tool for AIS treated conservatively with PSSE and for the patients’ clinicians.

## 1. Introduction

In the existing literature for idiopathic scoliosis (IS), the following is widely reported: the common type of it [1,2], its 3-D character [3,4], the incidence of adolescent idiopathic scoliosis (AIS) [4], and how an early diagnosis is made performing the forward bending test, called Adam’s test [5]. Also, the curve that is named scoliosis according to the SRS is discussed [6,7]. Additionally, a plethora of publications report many possible genetic and epigenetic factors [4,8] as causes of its development.

Although scoliosis is considered as a harmless condition, evidence suggests that people with scoliosis are more prone to experiencing back and low-back pain [9,10]. Scoliosis causes multiple dysfunctions and appears to be a burden on healthcare, particularly when patients require extensive surgical treatment [11,12]. 

The application of Physiotherapeutic Specific Exercises for Scoliosis (PSEE) alone or with bracing is currently one of the therapeutic models for mild and moderate IS. 

As far as the definitions of the severity of scoliosis go, there is not full agreement on what is mild and moderate idiopathic scoliosis. Mild idiopathic scoliosis is characterized by a Cobb angle of more than 10 and less than 30 degrees [13], of more than 10 but less than 25 degrees [14], and of more than 10 but less than 20 degrees [4]. Moderate IS is characterized by a Cobb angle of 25–40 degrees, which is indicated for non-operative treatment [2,15] and a Cobb angle greater than 21 to 35 degrees [4]. We consider as mild curves those with a Cobb angle of greater than 10 but less than 20 degrees and as moderate those with a Cobb angle of greater than 21 to 35–40 degrees. 

SOSORT uses the term Physiotherapeutic Specific Exercises for Scoliosis (PSSE) for all approved schools and methods. Each method and school incorporates the SOSORT guideline principles and shares a common goal, that of stabilization, arresting progression of IS, and improving the quality of life of patients. The methodology of the PSSE must be based on scientific evidence and adjusted according to the type of the deformity of each patient [16,17]. Another protagonistic and high-priority common goal during the implementation of PSSEs, as recommended by SOSORT, is “three-dimensional self-correction” [4,18]. Self-correction can be defined as the best possible trunk alignment that a patient with scoliosis can achieve in the three planes and axes [19].

The existing PSSE schools that were created involve the following [17,20]:

- Schroth, Germany
- Lyon, France
- SEAS (Scientific Exercise Approach to Scoliosis), Italy
- BSPTS (Barcelona Scoliosis Physical Therapy School), Spain
- Side Shift, UK
- DoboMed, Poland
- FITS (Functional Individual Therapy of Scoliosis), Poland

In Greece, many of the above methods are applied for the rehabilitation of IS. To our knowledge, a specific questionnaire for PSSEs has not yet been published. In this report, a questionnaire is presented, which aims to evaluate the quality of life of children and adolescents with diagnosed IS, who are undergoing treatment with one of the above PSSE methods. 

## 2. Materials and Methods

### 2.1. The Questionnaire

For the development of the PSSEQ (Physiotherapeutic Scoliosis-Specific Exercises Questionnaire), we followed the recommended guidelines [21] for designing a questionnaire. It is based on a thorough literature review of the field of scoliosis and Physiotherapeutic Scoliosis-Specific Exercises (PSSEs), which are approved by the SOSORT organization [4], as well as on our experience regarding the needs of scoliotic children and adolescent patients in specialized physiotherapeutic centers, in our country. Permission and approval for conducting the research were requested and provided by the ethical committee of the Medical Association of Piraeus. In addition, because participants were minors, consent was provided by their parents for using data from their children who participated in the study.

The total number of the questions was chosen and categorized accordingly to the SOSORT guidelines and scientific articles of IS. The development of questions was carried out by the authors who subsequently divided the questionnaire into the following 8 domains: (1) physical functioning (question numbers 1, 5, 6, 20, 21, 23, 24, 25, 26, 34, 35, 40), (2) self-image (question numbers 2, 47, 49, 50), (3) Physiotherapeutic Scoliosis-Specific Exercises (PSSEs) (question numbers 3, 19, 27, 28, 32, 37, 38, 39), (4) psychosocial functioning (question numbers 4, 7, 22, 29, 36, 41, 42, 44, 45, 46, 48, 51), (5) cognitive functioning (question numbers 11,18, 30, 52,53), (6) compliance (question numbers 8, 9, 10, 12, 13, 14, 15, 16), (7) motivation (question numbers 17, 33), and (8) pain (question numbers 31, 43). 

The questionnaire consists of 53 questions. There are 37 with positive meaning and 15 with negative meaning and 1 multiple choice question. Furthermore, 6 open-ended questions were included related to the PSSEs and the questionnaire. In the scored items, a Likert scale of 1 -5 was used. More specifically, responses 1–5 correspond to 5 = Strongly agree and 1 = Strongly disagree. An additional edit was made to the wording of the questions so that they could correspond meaningfully to these responses. 

The first sheet of the questionnaire includes questions about the date of data collection, demographic characteristics, first name and surname, father’s and mother’s names, gender, date of birth, address, contact details, height and weight in kilos to calculate BMI, right or left handedness, date of menarche for females, hair and eye color, and type of scoliosis. Data to be filled in by the researcher include the Scoliometer angle, Cobb angle, Risser sign, (Formetric—scoliosis angle), type of treatment (exercise or both exercise and bracing). 

A pre-final PSSE questionnaire was developed and a pilot study was conducted, which was completed by 16 participants with AIS. We asked the 16 participants to complete this questionnaire so that we could calculate Cronbach’s Alpha based on the results in their responses. The total sample responses to the questions were entered in SPSS and for each domain, the minimum and maximum effect (floor and ceiling effect), defined as the percentage of participants showing the minimum and maximum possible scores, respectively, were calculated. Results at the minimum and maximum effect exceeding 15% were considered statistically significant. The floor effect was 53/265 and the ceiling effect was 265/265, respectively. Cronbach’s Alpha was calculated as >0.7, which is considered a strong correlation–standard-deviation result between responses. Internal consistency of the questionnaire was evaluated by calculating the Cronbach’s Alpha. The initial number of questions was 64; thus, 11 were excluded from the study or combined with the existing questions just to become more specific and easier for the patients to understand. Thus, the final PSSE questionnaire consists of 53 questions, and 6 “open” questions; see Supplementary Materials. The original questionnaire for PSSEs was developed in the Greek language and was coined QPSSE.

### 2.2. Study Population

The inclusion criteria for participating in this study are the following: (1) patients should be from 10 to 18 years old, (2) patients should have been diagnosed with mild or moderate AIS, (3) patients should be able to speak and read in Greek, and (4) patients should be undergoing or have undergone in the past Physiotherapeutic Scoliosis-Specific Exercises (PSSEs) for a period of at least 2 months. 

The exclusion criteria were mental health problems, a low level of communication, congenital, neurological, or another type of scoliosis, and/or having or had surgery for scoliosis.

Eighty patients qualified and were included in the study. For their characteristics, see below in Section 3.1.

### 2.3. Psychometric Evaluation

The PSSE questionnaire was evaluated for the following psychometrics: reliability, validity, and floor and ceiling effects. Reliability was assessed by analyzing internal consistency and test–retest reliability. Internal consistency was determined using the Cronbach’s Alpha. The intraclass correlation coefficient (ICC) was calculated to measure the test–retest reliability. The patients completed the questionnaire twice at an interval of 4–7 days for the measuring of test–retest reliability. Convergent validity was compared against the critical value of Pearson’s correlation. Divergent validity was evaluated by analyzing the answers of the patients of the PSSEQ and their characteristics (e.g., Risser sign, Formetric, Cobb angle, gender, etc.) using Pearson’s or Spearman correlation coefficient. The IBM SPSS Statistics v.25 was used for the statistical analysis.

### 2.4. Materials

#### 2.4.1. Scoliometer Measurement (ATR)

Using a Scoliometer, the angle of trunk rotation (ATR) was assessed [18]. 

#### 2.4.2. Formetric 4D-DIERS

The Formetric 4D DIERS is a scanning system with a light projector, which scans the back of the patient. This system is connected to a computer that analyzes the data and provides information about the posture of the body, spinal curves (frontal, lateral), pelvic position, vertebral rotation, and muscle imbalance [19].

#### 2.4.3. BMI

Body mass index (BMI) involves a method that calculates body fat according to height and weight in females or males. A normal BMI range is between 18.5 and 24.9 [22]. 

#### 2.4.4. Cobb Angle

The Cobb angle measurement is used for calculating spine curvatures in the frontal plane. A radiograph is necessary in order for the Cobb angle to be measured. This method is used so as to determine the upper/lower-end vertebras (UEV/LEV) on the radiograph; then, a vertical line at the, respectively, upper/lower-end vertebra endplate lines is necessary (UEVEL/LEVEL), and the included angle of the two vertical lines is the Cobb angle [23].

#### 2.4.5. Risser Sign

The Risser sign is used by clinicians in order to assess the skeletal maturity of a human. The Risser sign is determined using the iliac apophysis from radiographs and is classified in 6 stages (0–5 Risser). It is often used for the evaluation of adolescent idiopathic scoliosis and for the selection of its treatment [24].

#### 2.4.6. Demographics

Demographics are characteristics of a population and are often evaluated for a statistical analysis. Some of them are age, gender, ethnicity, education, geographic location, etc. In this study, age, gender, and color of hair and eyes were used in the statistical analysis.

## 3. Results

Time needed to complete the PSSEQ was about 10–11 min. 

Eighty patients were included in the study and 21 test–retests were completed in a period of 14 months.

### 3.1. Descriptive Statistics



MeanStandard DeviationAge (yrs)
16.57.1BMI (Kg/m^2^)
19.892.75Cobb angle (degrees)
36.14.08Risser sign
4.61.6Age at menarche
12.11.1

CountColumn N %MenarcheNo1218.2%
Yes5481.8%SexMale1113.8%
Female6986.3%BraceNo1925.0%
Yes5775.0%Dominant handRight6788.2%
Left911.8%In general, how would you describe the state of your physical health?Not good at all11.3%
Somewhat good11.3%
Moderately good2025.0%
Quite good4151.3%
Very good1721.3%In general, how would you rate your appearance?Not good at all00.0%
Somewhat good22.5%
Moderately good1721.3%
Quite good3948.8%
Very good2227.5%In general, how would you describe your experience with PSSE?Not good at all00.0%
Somewhat good11.3%
Moderately good1113.8%
Quite good4252.5%
Very good2632.5%In general, how would you describe your mental health?Not good at all00.0%
Somewhat good33.8%
Moderately good1113.8%
Quite good4050.0%
Very good2632.5%In general, how would you rate your posture during the day?Not good at all22.5%
Somewhat good56.3%
Moderately good2936.3%
Quite good3645.0%
Very good810.0%In general, how would you rate your endurance?Not good at all33.8%
Somewhat good67.5%
Moderately good1316.3%
Quite good2632.5%
Very good3240.0%In general, how would you describe your relationship with the people close to you?Not good at all00.0%
Somewhat good00.0%
Moderately good56.3%
Quite good1721.3%
Very good5872.5%In this time period, I do PSSE alone at home every day.Never11.3%
Almost never56.3%
Sometimes2835.0%
Almost constantly3240.0%
Constantly1417.5%In this time period, I keep a PSSE diary.Never5163.8%
Almost never1012.5%
Sometimes78.8%
Almost constantly56.3%
Constantly78.8%In this time period, I do PSSE regularly and according to the physiotherapist’s instructions.Never33.8%
Almost never11.3%
Sometimes2227.5%
Almost constantly2632.5%
Constantly2835.0%In this time period, among my daily activities is trying to make sure that my body weight is better distributed on the right and left side.Never22.5%
Almost never67.5%
Sometimes2936.3%
Almost constantly3948.8%
Constantly45.0%I will do PSSE at home even if I’m tired.Not true at all67.5%
Somewhat true1620.0%
Almost true1721.3%
True enough3138.8%
Absolutely true1012.5%I will do PSSE at home even if I’m in a bad mood.Not true at all1012.5%
Somewhat true1417.5%
Almost true1923.8%
True enough2227.5%
Absolutely true1518.8%I will do PSSE at home even if I don’t have time.Not true at all1215.0%
Somewhat true2632.5%
Almost true2328.8%
True enough1417.5%
Absolutely true56.3%I will do PSSE at home even if I’m on vacation.Not true at all2733.8%
Somewhat true1923.8%
Almost true1620.0%
True enough1113.8%
Absolutely true78.8%I will do PSSE at home even if the physiotherapist is not with me.Not true at all67.5%
Somewhat true810.0%
Almost true1518.8%
True enough1822.5%
Absolutely true3341.3%MotivationI try to do the PSSE because if I don’t, I will feel guilty.45.0%
I try to do PSSE because I don’t want to upset some people who are very important to me.1620.0%
PSSE are worth the effort and time I spend because of the positive effects on my body.3746.3%
I do PSSE because I feel great satisfaction when I achieve the goals I set during each treatment.1417.5%
I do PSSEs because they really help me feel good about myself and my life.911.3%The PSSEs are important in treating scoliosis.Not true at all00.0%
Somewhat true00.0%
Almost true67.5%
True enough2936.3%
Absolutely true4556.3%The PSSEs are very useful for me.Not true at all00.0%
Somewhat true00.0%
Almost true1316.3%
True enough3341.3%
Absolutely true3442.5%The PSSEs help me because I feel more energetic.Not true at all1012.5%
Somewhat true1012.5%
Almost true2632.5%
True enough2126.3%
Absolutely true1316.3%The PSSEs help me breath better.Not true at all1012.5%
Somewhat true1215.0%
Almost true2430.0%
True enough2328.8%
Absolutely true1113.8%The PSSEs put me in a difficult position because I waste a lot of my free time.Absolutely true1215.0%
True enough2733.8%
Almost true1215.0%
Somewhat true1620.0%
Not true at all1316.3%The PSSEs help me sleep better.Not true at all1822.5%
Somewhat true2328.8%
Almost true2936.3%
True enough78.8%
Absolutely true33.8%The PSSEs help me feel that my body is stronger and more stabilized.Not true at all56.3%
Somewhat true45.0%
Almost true1721.3%
True enough3645.0%
Absolutely true1822.5%The PSSEs are exhausting.Absolutely true1012.5%
True enough911.3%
Almost true2126.3%
Somewhat true2531.3%
Not true at all1518.8%The PSSEs help me have better endurance.Not true at all56.3%
Somewhat true1113.8%
Almost true3037.5%
True enough2632.5%
Absolutely true810.0%The PSSEs are easier when I do them in front of the mirror because I can see if I’m doing them right.Not true at all00.0%
Somewhat true11.3%
Almost true911.3%
True enough2936.3%
Absolutely true4151.3%The PSSEs are very difficult.Absolutely true1012.5%
True enough911.3%
Almost true2835.0%
Somewhat true1620.0%
Not true at all1721.3%The PSSEs make me feel good about myself.Not true at all33.8%
Somewhat true67.5%
Almost true2227.5%
True enough3543.8%
Absolutely true1417.5%The PSSEs help change my body for the better.Not true at all00.0%
Somewhat true22.5%
Almost true78.8%
True enough2936.3%
Absolutely true4252.5%The PSSEs help me feel less pain.Not true at all1316.3%
Somewhat true1923.8%
Almost true1518.8%
True enough1316.3%
Absolutely true2025.0%During the time I’m doing PSSEs, I see my body becoming more straight.Not true at all00.0%
Somewhat true00.0%
Almost true1215.0%
True enough3746.3%
Absolutely true3138.8%During the time I’m doing PSSEs, I try hard to do every exercise.Not true at all22.5%
Somewhat true67.5%
Almost true1620.0%
True enough3138.8%
Absolutely true2531.3%During the time I’m doing PSSEs, I feel that I can’t take a deep and satisfying breath.Absolutely true1923.8%
True enough2227.5%
Almost true1113.8%
Somewhat true1113.8%
Not true at all1721.3%During the time I’m doing PSSE, my chest feels tight and can’t “inflate” as much as it should. Absolutely true2531.3%
True enough1417.5%
Almost true1215.0%
Somewhat true1316.3%
Not true at all1620.0%During the time I’m doing PSSE, I prefer not to be seen by anyone.Absolutely true2430.0%
True enough1620.0%
Almost true911.3%
Somewhat true1620.0%
Not true at all1518.8%During the time I’m doing PSSE, I can straighten my body from a sitting position.Not true at all00.0%
Somewhat true67.5%
Almost true1012.5%
True enough2632.5%
Absolutely true3847.5%During the time I’m doing PSSE, I can straighten my body from a lying position.Not true at all56.3%
Somewhat true810.0%
Almost true911.3%
True enough3442.5%
Absolutely true2430.0%During the time I’m doing PSSE, I can straighten my body from a standing position.Not true at all22.5%
Somewhat true1012.5%
Almost true1620.0%
True enough2227.5%
Absolutely true3037.5%How often have you felt out of breath lately?Always2632.5%
Almost always2227.5%
Sometimes1215.0%
Almost never67.5%
Never1417.5%How often have you lately felt bad about yourself?Always1518.8%
Almost always2227.5%
Sometimes2632.5%
Almost never45.0%
Never1316.3%How often have you lately felt sad, down, or angry?Always56.3%
Almost always2126.3%
Sometimes4151.3%
Almost never78.8%
Never67.5%How often have you lately felt pain in your body?Always1518.8%
Almost always2025.0%
Sometimes2227.5%
Almost never1417.5%
Never911.3%How often have you lately felt that you have difficulties in making friends?Always2531.3%
Almost always1923.8%
Sometimes1215.0%
Almost never56.3%
Never1923.8%How often have you lately felt uncomfortable when you’re around people?Always1518.8%
Almost always2835.0%
Sometimes1316.3%
Almost never1316.3%
Never1113.8%It is true for me that I find it hard to open up to someone, especially as long as I’ve been dealing with PSSEs?Strongly agree2531.3%
Agree2126.3%
Neutral1215.0%
Disagree56.3%
Strongly disagree1721.3%It is true for me that I am not ashamed to do the PSSEs in front of people I consider close to me? Strongly disagree33.8%
Disagree810.0%
Neutral911.3%
Agree1620.0%
Strongly agree4455.0%It is true for me that my physical therapist is very friendly and supportive? Strongly disagree00.0%
Disagree11.3%
Neutral22.5%
Agree1721.3%
Strongly agree6075.0%It is true for me that my body’s curve is too obvious to others? Strongly agree1215.0%
Agree911.3%
Neutral2733.8%
Disagree2025.0%
Strongly disagree1215.0%It is true that when I meet someone, I worry about what they think about my appearance?Strongly agree1620.0%
Agree810.0%
Neutral2733.8%
Disagree1822.5%
Strongly disagree1113.8%It is true for me that it doesn’t matter how someone looks?Strongly disagree56.3%
Disagree911.3%
Neutral1215.0%
Agree3240.0%
Strongly agree2227.5%In general, how satisfied are you with the knowledge you gained through your experience with PSSE? Not at all00.0%
A little00.0%
Moderately1012.5%
Enough4657.5%
Very much2430.0%In general, how satisfied are you with your physical therapist?Not at all11.3%
A little00.0%
Moderately33.8%
Enough1620.0%
Very much6075.0%

### 3.2. Statistical Analysis

Based on the Kolmogorov–Smirnov goodness-of-fit test and Shapiro–Wilk test for normality, data did not follow the normal distribution; therefore, non-parametric tests were used for the statistical analysis. Pearson’s correlation coefficient was used for all correlations evaluated. *p* values of less than 0.05 were considered to be significant. Internal consistency was evaluated through the Cronbach’s Alpha method. Although there were very few missing values, accounting for 0.78% of the total values of the questionnaire, the expectation maximization likelihood algorithm was used to impute data. IBM^®^ SPSS^®^ Statistics Software v.25 was used for the analysis. 

### 3.3. Factor Analysis

The results of the content validity analysis demonstrated excellent reliability and content validity for the questionnaire, as summarized in Table 1.

### 3.4. Internal Consistency Reliability

Cronbach’s Alpha coefficients for the overall score were 0.84, exceeding the minimum recommended standard of 0.70 and indicating satisfactory internal consistency.

### 3.5. Item Convergent Validity

The criterion for item convergent validity was the correlation coefficient of each item of each domain with the domain scale variable. This value was compared against the critical value of Pearson’s r equal to 0.219, taking into account 78 degrees of freedom for each comparison with a *p* < 0.05. 

### 3.6. Item Divergent Validity

The criterion for item divergent validity was the correlation coefficient of each domain scale variable and the clinical continuous variables (age, BMI, Cobb angle). Except for the pain domain variable that was positively correlated with age, there was no other significant correlation found between the aforementioned variables, indicating the lack of relationship of the 53 measurements of the questionnaire with the clinical data.

### 3.7. Test–Retest Reliability

Test–retest reliability was assessed using Pearson’s correlation coefficient, r. Twenty-two subjects were re-evaluated one week after the first interview. The results of the questionnaire were used for 53 discrete bivariate correlations, 1 for each variable of the questionnaire. The results showed that there was perfect test–retest reliability in terms of achieving r values equal to 1 in all correlations. 

### 3.8. Floor and Ceiling Effects for the Overall Score

For the overall score, in the present study, 0% of patients scored at the floor and 0% scored at the ceiling. Therefore, there were no floor or ceiling effects for the overall score. Floor and ceiling effects for each domain are shown in Table 1. Floor and ceiling effects for each item are shown in Table 2.

The total values for all scale scores, as well as the range of possible scores, are shown in Table 3.

## 4. Discussion

The aim of this study was to develop a questionnaire that evaluates the quality of life in the Greek population with idiopathic scoliosis, who are undergoing Physiotherapeutic Specific Exercises for Scoliosis (PSSE), and the evaluation of its psychometrics. 

As it is described below, many other good questionnaires are used to assess scoliosis but a specific treatment for a PSSE questionnaire was not developed until the present. This makes the difference from other questionnaires developed to assess the QoL for IS. 

This treatment-specific questionnaire, the QPSSE, was created in order to determine how various parts of treatment of IS using PSSEs influence patients with AIS, and this is its strong point, which is not provided using other pertinent-to-IS questionnaires.

A final 53-item questionnaire was developed with eight domains. The eight domains of the final questionnaire were as follows: (1) physical functioning, (2) self-image, (3) Physiotherapeutic Scoliosis-Specific Exercises (PSSEs), (4) psychosocial functioning, (5) cognitive functioning, (6) compliance, (7) motivation, and (8) pain.

Previous studies have developed other questionnaires that evaluate similar aspects of patients with AIS after other treatments, either conservative or surgical. One questionnaire that evaluates the quality of life regarding AIS is the 22-item revised questionnaire of SRS (SRS-22R) that evaluates quality of life regarding AIS, especially after surgery treatment [25]. Another one is the Brace questionnaire (BrQ) that evaluates quality of life in populations with IS treated with a brace [26]. Also, Short-Form Health Survey 36 (SF-36) or Short-Form Health Survey 12 (SF-12) that evaluates Health-Related Quality of Life (HRQoL) [27,28] and the spinal appearance questionnaire (SAQ) aim to assess self-image in patients with AIS [29].

SRS-22-R is one of the most frequently used questionnaires for patients with scoliosis and includes 22 items that are divided in five domains: pain, self-image, function, mental health, and satisfaction with management [30]. QPSSE was divided into eight domains. Some of them were, also, pain, self-image, and psychosocial functioning. As it was mentioned before, SRS-22R was mainly intended for patients with AIS treated with surgery. However, to date, no questionnaires have been developed to evaluate the quality of life for patients with AIS, so SRS-22R was often used for evaluating general quality of life for these patients regardless of their treatment.

BrQ is a self-administrated questionnaire that evaluates the quality of life for patients with AIS who are treated with a brace. There is a 34-item Likert scale that consists of eight domains such as QPSSE general health perception, physical functioning (physical functioning, also, in QPSSE), emotional functioning (psychosocial functioning in QPSSE), self-esteem and aesthetics (self-image in QPSSE), vitality (motivation in QPSSE), school activity, bodily pain (pain in QPSSE), and social functioning (psychosocial functioning in QPSSE) [26].

SF-36 is a 36-item self-reported questionnaire and one of the most widely used Health-Related Quality of Life questionnaires. It is divided into eight sections: (1) vitality or energy (motivation in QPSSE), (2) physical functioning (also in QPSSE), (3) bodily pain (also in QPSSE), (4) general health perceptions, (5) physical role functioning, (6) emotional role functioning, (7) social role functioning (psychosocial functioning in QPSSE), (8) mental health or emotional wellbeing (cognitive functioning in QPSSE). SF-12 is a smaller version of SF-36. These questionnaires have also been used for patients with AIS in order to evaluate their general quality of life [27,28].

The 32-item SAQ is a questionnaire based on the Walter Reed Visual Assessment Scale (WRVAS) and it evaluates perception of spinal appearance by patients with AIS. After validation, a 20-item SAQ was developed and divided into nine domains, and three textual items about the most distressing aspects of deformity. The items of SAQ are divided into the following domains: general (three items), curve (one), prominence (two), trunk shift (two), waist (three), shoulders (two), kyphosis (one), chest (two), and surgical scar (one), and an extra three textual questions as it was mentioned. The SAQ has been widely used and culturally adapted into many languages for the assessment of the appearance in patients with AIS. The original Greek-QPSSE (has) also included a domain about the self-image of patients with AIS (four items) [31].

The PSSEQ results showed that there was perfect test–retest reliability in terms of achieving r values equal to 1 in all correlations and an overall score for internal consistency of 0.84, exceeding the minimum recommended standard of 0.70 and indicating satisfactory internal consistency. The Greek version of SRS-22 was shown to have three domains with a very satisfactory Cronbach’s α (pain, 0.85; mental health, 0.87; self-image, 0.83) and for two domains (function/activity, 0.72; satisfaction, 0.67), they were good. The intraclass correlation coefficient (ICC) was >0.70, demonstrating very satisfactory or excellent test/retest reliability [30]. The initial Greek BrQ was shown to have satisfactory internal consistency with a Cronbach’s Alpha of 0.82 [26]. The Greek version of SF-36 was found to have a Cronbach’s α > 0.70 [32]. The original English SAQ had good-to-excellent reliability (Spearman’s rho, 0.57–0.99) and high internal scale consistency (Cronbach’s Alpha > 0.7) [29].

Divergent validity in QPSSE was found with no other significant correlation between the aforementioned variables, indicating the lack of relationship of the 53 measurements of the questionnaire with the clinical data. Concurrent validity of SRS-22 was evaluated through its correlation with SF-36 domains analyzing Pearson Correlation Coefficients, and all correlations were found to be statistically significant [30]. Other cultural adaptations of SAQ evaluated convergent validity by correlating SAQ with the appearance domain of SRS-22R and divergent validity by correlating patients’ answers in SAQ with their characteristics, demonstrating good-to-excellent results [31,33,34,35,36] as in this study of the original Greek QPSSE. 

The original Greek version of QPSSE showed similar results with the Greek versions or other studies of SRS-22, BrQ, SF-36, or SF-12 and SAQ. All these questionnaires evaluate the quality of life for patients with AIS generally or after a treatment or assess Health-Related Quality of Life in general. Our study assessed the intervention after Physiotherapeutic Scoliosis-Specific Exercises, BrQ after bracing, and SRS-22 especially after surgery. However, further study of the QPSSE is needed in order to evaluate more psychometric properties, such as convergent validity or responsiveness. These tests would be necessary so as to determine if the PSSQ is responsive to changes. In order to assess convergent validity, it would be essential for other similar questionnaires or tools about the PSSEs and quality of life to be developed. Furthermore, a further study with a greater number of participants would potentially have better results in psychometrics in a questionnaire evaluating the quality of life in the Greek population with AIS.

This questionnaire is a significant tool for the clinicians and physical therapists who work using PSSEs for adolescents with IS, in order to evaluate their patients’ quality of life and interventions after exercises. This tool will provide clinicians with information about Greek patients with AIS so as to improve or change something in their treatment or intervention. It would be essential for this questionnaire to be translated into other languages too, so that other countries could have a tool available for evaluation of quality of life for patients who undergo PSSEs. 

A limitation of the application of this QPSSE may be considered as the time period of scoliosis exercise treatment, which is not clearly agreed upon and recommended on any curve type, and consequently the outcomes based on the generated data will need carefully conducted further studies including long-term follow ups.

## 5. Conclusions

In conclusion, the PSSE questionnaire was found to be reliable and valid for clinical use for patients with AIS treated conservatively with PSSEs or both PSSEs and a brace in the Greek population. 

## Figures and Tables

**Table 1:** The questionnaire domains and the results of tests of item convergent validity, item consistency reliability, and floor and ceiling effects.

Domain	Number of Items	Cronbach’s Alpha	Item Convergent Validity *	Floor Effects	Ceiling Effects
Physical functioning	12	0.452	75.0%	0 (0%)	0 (0%)
Self-Image	4	0.16	100.0%	0 (0%)	0 (0%)
PSSEs	8	0.514	87.5%	0 (0%)	0 (0%)
Psychosocial functioning	12	0.75	75.0%	0 (0%)	0 (0%)
Cognitive functioning	5	0.641	100.0%	0 (0%)	0 (0%)
Compliance	8	0.822	100.0%	0 (0%)	1 (1.25%)
Motivation	2	0.156	100.0%	0 (0%)	3 (3.8%)
Pain	2	0.313	50.0%	4 (5%)	2 (2.5%)
Total	53	0.836	79.2%	0 (0%)	0 (0%)

* Percentage of item–scale correlations ≥ 0.219.

**Table 2:** Floor and ceiling effects (percentage of respondents with minimum/maximum scale scores) for each item of the questionnaire.

No. of Item	Floor Effect	Ceiling Effect
1	1 (1.3%)	17 (21.3%)
2	0 (0%)	22 (27.5%)
3	0 (0%)	26 (32.5%)
4	0 (0%)	26 (32.5%)
5	2 (2.5%)	8 (10%)
6	3 (3.8%)	32 (40%)
7	0 (0%)	58 (72.5%)
8	1 (1.3%)	14 (17.5%)
9	51 (63.8%)	7 (8.8%)
10	3 (3.8%)	28 (35%)
11	2 (2.5%)	4 (5%)
12	6 (7.5%)	10 (12.5%)
13	10 (12.5%)	15 (18.8%)
14	12 (15%)	5 (6.3%)
15	27 (33.8%)	7 (8.8%)
16	6 (7.5%)	33 (41.3%)
17	4 (5%)	9 (11.3%)
18	0 (0%)	45 (56.3%)
19	0 (0%)	34 (42.5%)
20	10 (12.5%)	13 (16.3%)
21	10 (12.5%)	11 (13.8%)
22	12 (15%)	13 (16.3%)
23	18 (22.5%)	3 (3.8%)
24	5 (6.3%)	18 (22.5%)
25	10 (12.5%)	15 (18.8%)
26	5 (6.3%)	8 (10%)
27	0 (0%)	41 (51.3%)
28	10 (12.5%)	17 (21.3%)
29	3 (3.8%)	14 (17.5%)
30	0 (0%)	42 (52.5%)
31	13 (16.3%)	20 (25%)
32	0 (0%)	31 (38.8%)
33	2 (2.5%)	25 (31.3%)
34	19 (23.8%)	17 (21.3%)
35	25 (31.3%)	16 (20%)
36	24 (30%)	15 (18.8%)
37	0 (0%)	38 (47.5%)
38	5 (6.3%)	24 (30%)
39	2 (2.5%)	30 (37.5%)
40	26 (32.5%)	14 (17.5%)
41	15 (18.8%)	13 (16.3%)
42	5 (6.3%)	6 (7.5%)
43	15 (18.8%)	9 (11.3%)
44	25 (31.3%)	19 (23.8%)
45	15 (18.8%)	11 (13.8%)
46	25 (31.3%)	17 (21.3%)
47	3 (3.8%)	44 (55%)
48	0 (0%)	60 (75%)
49	12 (15%)	12 (15%)
50	16 (20%)	11 (13.8%)
51	5 (6.3%)	22 (27.5%)
52	0 (0%)	24 (30%)
53	1 (1.3%)	60 (75%)

**Table 3:** Mean values and ranges for each item of the questionnaire.

No. of Item	Mean Value	Range
1	3.9	1–5
2	4.0	2–5
3	4.2	2–5
4	4.1	2–5
5	3.5	1–5
6	4.0	1–5
7	4.7	3–5
8	3.7	1–5
9	1.8	1–5
10	3.9	1–5
11	3.5	1–5
12	3.3	1–5
13	3.2	1–5
14	2.7	1–5
15	2.4	1–5
16	3.8	1–5
17	3.1	1–5
18	4.5	3–5
19	4.3	3–5
20	3.2	1–5
21	3.2	1–5
22	2.9	1–5
23	2.4	1–5
24	3.7	1–5
25	3.3	1–5
26	3.3	1–5
27	4.4	2–5
28	3.3	1–5
29	3.6	1–5
30	4.4	2–5
31	3.1	1–5
32	4.2	3–5
33	3.9	1–5
34	2.8	1–5
35	2.8	1–5
36	2.8	1–5
37	4.2	2–5
38	3.8	1–5
39	3.9	1–5
40	2.5	1–5
41	2.7	1–5
42	2.9	1–5
43	2.8	1–5
44	2.7	1–5
45	2.7	1–5
46	2.6	1–5
47	4.1	1–5
48	4.7	2–5
49	3.1	1–5
50	3.0	1–5
51	3.7	1–5
52	4.2	3–5
53	4.7	1–5

## Data Availability

Data are available on demand.

## Supplementary Materials

The following supporting information can be downloaded at: https://www.mdpi.com/article/10.3390/jcm13020318/s1, Questionnaire for Physiotherapeutic Specific Scoliosis Exercises (QPSSE).

## Abbreviations

AIS = Adolescent Idiopathic Scoliosis; IS = Idiopathic Scoliosis; ATR = Angle of Trunk Rotation; IIS = Infantile Idiopathic Scoliosis; JIS = Juvenile Idiopathic Scoliosis; PSSEs = Physiotherapeutic Scoliosis-Specific Exercises; TA = Truncal Asymmetry.
