# Supplementary material for: A Questionnaire of Physiotherapeutic Specific Exercises of Scoliosis—QPSSE
Source: J Clin Med. 2024 Jan 5;13(2):318. doi: 10.3390/jcm13020318 (PMC10816487; doi:10.3390/jcm13020318)
Supplement: Supplementary file 1 [file jcm-13-00318-s001.zip › jcm-2755920-supplementary.pdf]

QUESTIONNAIRE FOR PHYSIOTHERAPEUTIC SPECIFIC SCOLIOSIS EXERCISES (QPSSE)

(ATTENTION: this page is to be completed by the physiotherapist/health promoter and not by the child being examined)

Date of filling in the questionnaire: ….. / ….. /202…..

Name: ………

Surname: ………………………………………………………………………………………………………

Father's Name: …………………………………………………………………………………………………

Sex: …….

Date of Birth:…………………………………………………………………………………………………

Address :……

Contact phone number:……………………………………………………………………………………

Email: …….

Height in cm:…………………………………………………………………………………………………

Weight in kg: …………………………… (BMI): ……………………………………………………………

Left-handed/Right-handed? ……………………………………………………………………………

For girls: Exact date the menstruation started: ……………………………………………………

Hair color: ……………………………………………………………………………………………………

Eye color: ……………………………………………………………………………………………………

Type of scoliosis: …………………………………………………………………………………………

Standing forward bend test- Scoliometer in degrees- Hump:

/ *scoliometer right*……… / thoracic………. /thoracolumbar…………ς/ lumbar……….

/ scoliometer left ……/ thoracic………. /thoracolumbar……../ lumbar…………

Seated forward bend test - Scoliometer in degrees- Hump :

/ *scoliometer right*: /thorasic………/thoracolumbar……………/lumbar……..

/ *scoliometer left* /thorasic………/thoracolumbar……………lumbar……..

Cobb Angle: **…………………………………………………………………………………………**

Risser Sign :……………………………………………………………………………………………………

Formetric: (if there is any please attach it)

Treatment (wearing brace?)

……
………………………………….

Which method of Physiotherapeutic Specific Scoliosis Exercises is being used: …………………………………………………………………………………

QUESTIONNAIRE FOR PHYSIOTHERAPEUTIC SPECIFIC SCOLIOSIS EXERCISES (PSSE)

Thank you for completing this questionnaire. In doing so please respond to the questions freely and truly for there are no right or wrong answers in the case of Physiotherapeutic Specific Scoliosis Exercises (PSSE).

Instructions: -Please read each question carefully. -Mark with an x the answer that suits you best, as in the example:

example		Absolutely true	True enough	Almost true	A little true	Not true at all
1.	PSSE are very useful for me		**x**			

	in general	Not good at all	Somewhat good	Moderately good	Quite good ~~enough~~	Very good
1.	..πως θα περιέγραφες την κατάσταση της σωματικής σου υγείας;/ how would you describe the state of your physical health?					
2.	..πως θα βαθμολογούσες την εξωτερική σου εμφάνιση;/ how would you rate your appearance?					
3.	..πως θα περιέγραφες την εμπειρία σου με τις ΕΦΑΣ;/ how would you describe your experience with PSSE?					
4.	..πως θα περιέγραφες τη ψυχική σου υγεία;/ How would you describe your mental health?					
5.	..πως θα βαθμολογούσες τη στάση του σώματος σου μέσα στη μέρα;/ How would you rate your posture during the day?					
6.	..πως θα βαθμολογούσες την αντοχή σου;/how would you rate your endurance?					
7.	..πως θα περιέγραφες τη σχέση σου με τα κοντινά σου άτομα;/ how would you describe your relationship with the people close to you?					

	Αυτόν τον καιρό../ This time period	Ποτέ/ Never	Σχεδόν ποτέ/ Almost never	Μερικές φορές/ Sometimes	Σχεδόν συνέχεια/Almost constantly	Συνέχεια/ Constantly
8.	..κάνω κάθε μέρα τις ΕΦΑΣ μόνος/η μου στο σπίτι/ I do PSSE alone at home every day					
9.	..κρατάω ημερολόγιο για τις ΕΦΑΣ/ I keep a PSSE diary					
10.	..κάνω τις ΕΦΑΣ τακτικά και σύμφωνα με τις οδηγίες του φυσικοθεραπευτή/ I do PSSE regularly and according to the physiotherapist's instructions					
11.	..στα πράγματα που κάνω μέσα στην μέρα φροντίζω το βάρος του σώματος μου να μοιράζεται καλύτερα δεξιά και αριστερά/ among my daily activities is trying to, I make sure that my body weight is better distributed on the right and left site					

Θα κάνω κανονικά τις ΕΦΑΣ στο σπίτι ακόμα και αν../I will do PSSE at home even if..		Καθόλου αληθές/ Not true at all	Λίγο αληθές/ Somewhat true	Σχεδόν αληθές/Almost true	Αρκετά αληθές/ True enough	Απολύτως αληθές/Absolutely true
12	..είμαι κουρασμένος/η/ I'm tired					
13	..είμαι κακόκεφος/η/ I'm in a bad mood					
14	..δεν έχω χρόνο/ don't have time					
15	..είμαι σε διακοπές/ I'm on vacation					
16	..δεν είναι μαζί μου ο φυσικοθεραπευτής/the physiotherapist is not with me					

17. Ποια από τις παρακάτω προτάσεις σας εκφράζει περισσότερο;/ Which one of the following sentences best fits your case? (Επιλέξτε <u>μόνο μία</u> απάντηση και σημειώστε με **x** το αντίστοιχο κουτάκι)/ (Choose only one answer and check the corresponding box with **x**)		
	α)	Προσπαθώ να κάνω τις ΕΦΑΣ γιατί άμα δεν τις κάνω θα νιώθω ενοχές./I try to do the PSSE because if I don't I will feel guilty
	β)	Προσπαθώ να κάνω τις ΕΦΑΣ γιατί δε θέλω να στεναχωρήσω κάποια άτομα που είναι πολύ σημαντικά για εμένα./ I try to do PSSE because I don't want to upset some people who are very important to me.
	γ)	Οι ΕΦΑΣ αξίζουν τον κόπο και το χρόνο που αφιερώνω σε αυτές γιατί έχουν θετικά αποτελέσματα στο σώμα μου./ PSSE are worth the effort and time I spend because of the positive effects on my body.
	δ)	Κάνω τις ΕΦΑΣ γιατί νιώθω μεγάλη ικανοποίηση όταν πετυχαίνω τους στόχους που θέτω στη διάρκεια κάθε θεραπείας./ I do PSSE because I feel great satisfaction when I achieve the goals I set during each treatment.
	ε)	Κάνω τις ΕΦΑΣ γιατί πραγματικά με βοηθούν στο να νιώθω όμορφα με τον εαυτό μου και τη ζωή μου. I do PSSE because they really help me feel good about myself and my life.

Οι ΕΦΑΣ.. /The PSSEs		Καθόλου αληθές/Not true at all	Λίγο αληθές/ Somewhat true	Σχεδόν αληθές/Almost true	Αρκετά αληθές/ True enough	Απολύτως αληθές/Absolutely true
18.	..είναι σημαντικές για την αντιμετώπιση της σκολίωσης/ are important in treating scoliosis					
19.	..είναι πολύ χρήσιμες για εμένα/ are very useful for me					
20.	..με βοηθούν γιατί νιώθω πως έχω περισσότερη ενέργεια/ help me because I feel more energetic					
21.	..με βοηθούν να αναπνέω καλύτερα/ help me breathing better					
22.	..με φέρνουν σε δύσκολη θέση γιατί "τρώνε" πολύ από τον ελεύθερο μου χρόνο/they put me in a difficult position because I waste a lot of my free time					

2.3	..με βοηθούν να κάνω καλύτερο ύπνο/ help me sleep better					
24.	..με βοηθούν να νιώθω το σώμα μου πιο δυνατό και σταθερό/ help me ~~to~~ feel my body stronger and more stabilized					
25.	..είναι εξαντλητικές/ are exhausting					
26.	..βοηθούν να έχω καλύτερη αντοχή/ help me ~~to~~ have better endurance					
27.	..είναι πιο εύκολες όταν τις κάνω μπροστά από τον καθρέφτη γιατί μπορώ να βλέπω άμα τις κάνω σωστά/ are easier when I do them in front of the mirror because I can see if I'm doing them right					
28.	..είναι πολύ δύσκολες/ are very difficult					
29.	..με κάνουν να νιώθω καλά με τον εαυτό μου/ make me feel good about myself					
30.	..βοηθούν στο να αλλάξει το σώμα μου προς το καλύτερο/ help change my body for the better					
31.	..βοηθούν στο να πονάω λιγότερο/ help me feel less pain					

Την ώρα που κάνω τις ΕΦΑΣ../ the time I'm doing PSSE		Καθόλου αληθές/ Not true at all	Λίγο αληθές/ Somewhat true	Σχεδόν αληθές/ Almost true	Αρκετά αληθές/ True enough	Απολύτως αληθές/Absolutely true
32.	..βλέπω τον κορμό μου να γίνεται πιο ίσιος/ I see my body becoming more straight					
33.	..προσπαθώ σκληρά να καταφέρω κάθε άσκηση/ I try hard to do every exercise					
34.	..νιώθω ότι δεν μπορώ να πάρω μια βαθιά και ικανοποιητική ανάσα/ I feel that I can't take a deep and satisfying breath					
35.	..νιώθω το στήθος μου σφιχτό και δεν μπορεί να "φουσκώσει" όσο πρέπει/ my chest feels tight and can't "inflate" as much as it should					
36.	..προτιμώ να μην με βλέπει κάποιος άλλος/I prefer not to be seen by anyone ~~else~~					
37.	....μπορώ να διορθώσω το σώμα μου από καθιστή θέση/I can straighten my body from a sitting position					
38.	..μπορώ να διορθώσω το σώμα μου από ξαπλωμένη θέση/I can straighten my body from a lying position					

39.	..μπορώ να διορθώσω το σώμα μου από όρθια θέση/ I can straighten my body from a standing position					

Πόσο συχνά το τελευταίο καιρό έχεις νιώσει../ How often have you lately felt...		Ποτέ/ Never	Σχεδόν ποτέ/ Almost Never-Rarely	Μερικές φορές/ Sometimes	Σχεδόν συνέχεια/ Almost Always	Συνέχεια/ Always
40.	.. μην σου "φτάνει" η ανάσα;/ being out of breath?					
41.	..άσχημα για τον εαυτό σου;/bad about yourself?					
42.	..λυπημένος/η, άκεφος/η ή νευριασμένος/η;/sad, down, or angry ?					
43.	..πόνο στο σώμα σου;/pain in your body?					
44.	..ότι δυσκολεύεσαι να κάνεις φίλους;/that you have difficulties in making friends?					
45.	..άβολα όταν βρίσκεσαι με κόσμο;/uncomfortable when you're around people?					

Ισχύει για εμένα ότι../It is true for me that..		Απολύτως διαφωνώ/ Strongly disagree	Αρκετά διαφωνώ/ Disagree	Ούτε διαφωνώ, ούτε συμφωνώ/ Neutral	Αρκετά συμφωνώ/ Agree	Απολύτως συμφωνώ/ Strongly agree
46.	Μου φαίνεται δύσκολο να ανοιχτώ σε κάποιον/α, ειδικά όσο καιρό ασχολούμαι με τις ΕΦΑΣ/ I find it hard to open up to someone, especially as long as I've been dealing with PSSE					
47.	Δεν ντρέπομαι να κάνω τις ΕΦΑΣ μπροστά σε άτομα που θεωρώ κοντινά μου/ I am not ashamed to do the PSSE in front of people I consider close to me					
48.	Ο/Η φυσικοθεραπευτής/τρια μου είναι πολύ φιλικός και υποστηρικτικός/ My physical therapist is very friendly and supportive					
49.	Η καμπύλη που κάνει το σώμα μου παραείναι εμφανής στους άλλους/ My body’s curve is too obvious to others					
50.	Όταν γνωρίζω κάποιον/α ανησυχώ για το τι μπορεί να σκέφτονται για εμένα και την εξωτερική μου εμφάνιση/ When I meet someone I worry about what they might think of me and my appearance					
51.	Δεν έχει σημασία το πως μοιάζει εξωτερικά κάποιος/α/					

	It doesn't matter what someone looks like on the outside					

Σε γενικές γραμμές../ In general		Καθόλου/ Not at all	Λίγο/ A little	Μέτρια/ Moderately	Αρκετά/ Enough	Πάρα πολύ/ Very much
52.	..πόσο είσαι ικανοποιημένος/η είσαι από τις γνώσεις που απέκτησες μέσω της εμπειρίας σου με τις ΕΦΑΣ;/ how satisfied are you with the knowledge you gained through your experience with PSSE?					
53.	..πόσο ευχαριστημένος είσαι από τον/την φυσικοθεραπευτή/τρια σου;/ how satisfied are you with your physical therapist?					

54. Τι θα ήθελες να ήταν διαφορετικό σε όλη την εμπειρία που είχες τώρα με τις ΕΦΑΣ;
What would you like to have been different in the whole experience you have had so far with PSSE?

55. Πως θα σου φαινόταν αν σου έλεγαν ότι πρέπει να συνεχίσεις τις ΕΦΑΣ μέχρι να ενηλικιωθείς;
How would you feel if you been told that you must continue PSSE until you reach adulthood?

56. Τι πιστεύετε ότι θα βοηθούσε στο να αλλάξει το ερωτηματολόγιο προς το καλύτερο;
What changes do you think would improve this questionnaire?

57. Σας κούρασε το ερωτηματολόγιο; (Παρακαλώ αιτιολογήστε)
Have you felt this was tiresome? (Please explain)

58. Θα προτιμούσατε να **μην** απαντήσετε σε κάποιες από τις ερωτήσεις; Σε ποιες; (Παρακαλώ καταγράψτε τα νούμερα των ερωτήσεων)
Would you rather not answer some of the questions? which ones? (Please write down the numbers of the questions)

59. Θα θέλατε να σχολιάσετε κάτι επιπλέον;
Would you like to comment on anything else?

Ευχαριστούμε πολύ για την συμμετοχή σας !

Thank you very much for your participation!
